# Jefferson Medical College

**Published:** 1843-05-13

**Authors:** H. T. Child


### JEFFERSON MEDICAL COLLEGE.

### CLINIC OF PROFESSOR MÜTTER.

*Dispensary of Jefferson Medical College, Jan.* 25, 1843.

(Reported by H. T. Child.)

### LECTURE V.

CASE—*Gelatinous Polypus of the Nose—Operation with forceps.*

Professor M. remarked that when a tumor, especially if it be of a pyriform shape, forms within any of the internal cavities which are lined by mucous membrane, it is termed polypus. It takes its name from an erroneous idea, for a long time entertained, that it had roots or feet similar to those of the sepia or marine polypus, by which it attaches itself to the neighbouring parts.

By far the most common variety of polypus is that which sprouts from the mucous membrane of the nose, and of which there are several varieties. We often, however, meet with similar tumors in the cavity of the antrum, frontal sinuses, larynx, pharynx, œsophagus, meatus auditorius, uterus, vagina and rectum.

*Location.*—Nasal polypi may occupy any portion of the nostrils, but they usually grow from the upper spongy bones and middle meatus, and for the most part select the outer surface or wall of the cavity. So common, indeed, is it for this portion of the nose to be the seat of the root of the tumor, that Sir A. Cooper declares he has never seen it springing from the septum narium. Sharp, S. Cooper, Cheleus and others, make the same statement; but I have met with an exception to this law in the person of a patient sent me by my friend Dr. Stokes, of Moorestown, N. J.; and Sir Cæsar Hawkins refers to a similar case. The tumor may, therefore, originate in the mucous membrane covering the septum, although, in by far the greater number of cases, it is attached elsewhere.

*Form.*—The shape of these tumors varies in different cases, inasmuch as it depends almost entirely upon the form of the cavity in which the new growth is contained. Usually in the commencement they are pyriform, oval, or round, but they may become flattened, elongated, lobulated, and finally so irregular as to defy comparison or description.

In nasal polypi where the tumor is single, and points either *forwards* or *backwards*, it is usually pyriform, and flattened by lateral pressure. When, on the other hand, it is composed of several cells, as in the vesicular polypus, its form is irregular and its surface rough: and when it assumes the malignant type, or is malignant from its origin, and, as is usually the case, is fibrous in texture and of large size, it is smooth on the surface, irregular in shape, and attached by a very extensive base, or by false membrane, to the surrounding parts.

*Number.*—There is great variety in the number of the tumors. In most cases, especially when the polypus is vesicular or gelatinous, we may expect to find more than one; often ten, fifteen, or even twenty may be detected, all held together by loose cellular tissue, and presenting, as a mass, very much the appearance of the ovarium or egg-bag of a fowl: when this is the case one or two large polypi present either at the anterior or posterior opening of the nostril, and prevent the detection of the smaller ones; but as soon as they are removed the latter make their appearance, and often prove a source of great annoyance to one not accustomed to operating, and who has probably told the patient that one or two introductions of the forceps will be sufficient to eradicate the whole disease. They must all be removed, however, if possible; for if one be allowed to remain it becomes the germ of a new collection, or may itself acquire a large size, and thus give rise to the necessity of a subsequent operation.

*Size.*—There is also great diversity in size among these nasal polypi; often in the *vesicular form* they are not larger, when first observed, than a small pea, but a number of these, by being attached to each other, may form a mass of considerable size. The gelatinous polypus is usually larger, being from an inch to two inches in length, by an inch or more in width; while the fibrous, although small in its commencement, often acquires an immense volume, destroying as it enlarges, the bones and cartilages by which it is surrounded, extruding the eyeball from its socket, shutting up the fauces, and requiring for its removal one of the most terrific operations ever devised by the surgeon.

*Consistence.*—These tumors also vary in consistence and texture, and upon this fact one of the classifications of the disease is based. Thus we speak of the *vesicular* or *mucous* polypus, the *gelatinous* (included by Boyer, along with the vesicular, under the single term of *mucous*,) the *fleshy*, the *fibrous*, the *scirrhous*, the *cartilaginous*, and the *osseous*.

*Colour.*—They vary likewise in colour; the *vesicular* and *gelatinous* being semi-transparent, bluish or whitish, or pale yellow or gray; the fleshy, red or pink; while the fibrous and schirrous are either pale or reddish.

*Termination.*—They vary also in their tendencies or results; and from this circumstance Mr. Pott particularly urged the doctrine that certain polypi are originally benign, whilst others are malignant. John Bell, whose chief pleasure seems to have been in exciting controversy, and throwing ridicule upon his professional brethren, declares that such an opinion is absurd, inasmuch as all polypi are benign in their commencement, and that, as they increase in size, *all* will terminate, if left to themselves, in the same terrible series of phenomena which belong to the malignant form of the disease. The opinion of Pott, however, is now generally adopted, and I shall take it as the basis of my classification. We may divide all nasal polypi into two groups:

1st. Those which are originally benign, but which may become malignant in process of time.

2d. Those which are originally malignant, and which, depending as they generally do, upon some constitutional taint, run their course rapidly, and invariably produce the most terrible results.

Under the first head I include—1st, the vesicular; 2d, the gelatinous; 3d, the fleshy; 4th, the fibrous; and 5th, the hard polypi.

Under the second—1st, the cancerous of mucous membranes; 2d, the medullary or hæmatoid; 3d, the schirrous.

Polypi have also been divided, according to their location, into *internal* and *external.*

*Causes.*—The precise causes of the development of nasal polypus have not, as yet, been clearly determined. In most cases it appears to be a strictly local disease, and susceptible of cure by local means alone. In others it evidently depends upon some constitutional affection, and is only to be eradicated by the employment of constitutional as well as topical remedies.

Many local causes, such as picking the nose, taking snuff, mechanical injuries, chronic inflammation of the Shneiderian membrane, ozena, ulcers, scrofula, repeated attacks of cold in the head, &c. &c., have all been cited as directly predisposing to the occurrence of polypus. But, unless chronic inflammation is produced by the first mentioned causes, it seems to me to exert little or no influence; when, however, this action is excited, there can be no question of its laying the foundation of the polypus growth. I have over and again seen chronic inflammation and ozena followed by polypus; and I have also known dyspepsia, by causing chronic inflammation of the mucous membrane of the fauces and nares, give rise to the complaint. Abernethy, Hawkins and others, make the same observation.

*General Symptoms.*—The presence of these tumors in the nostrils, whatever may be their nature, will always give rise to certain general symptoms. The patient usually complains at first of a stuffing of the head, as in common catarrh, which is also accompanied by a secretion of mucous, as in the latter complaint. This attracts little or no attention for some time, but by degrees he finds that he cannot smell so acutely as usual, that his voice is altered, that he is unable to breathe readily when the mouth is closed, and that his taste is also impaired. By and by the tears, provided the polypus points toward the anterior nares, pour over the cheek, in consequence of the obstruction of the ductus ad nasum, and there is likewise an unnatural fulness about the nose.

When the tumor is directed toward the fauces there will be difficulty in deglutition; and when it presses upon the Eustachian tube deafness will result.

Should the individual fail to receive proper assistance at this time, other symptoms are added to those already enumerated; pain of a dull character, unless the disease be malignant, when it is usually sharp and lancinating, now sets in; the patient is drowsy, from the circulation in the brain being interfered with; he is also chilly and indisposed to exertion; the bones of the nose and face enlarge; his skin becomes sallow; his appetite leaves him; there is a discharge of sanious fetid matter from the nostril; sometimes a sudden gush of blood from the opening of a vessel takes place, and the disposition to coma increases. In a few weeks more the poor wretch is reduced to the most terrible condition; in constant agony; with a fetor about him that disgusts and sickens both himself and his friends; with a loathsome secretion constantly poured into his throat, impregnating each morsel of food that he takes; with nearly all his faculties of relation lost, he exhibits a most deplorable example of misery and distress; at length, in mercy, coma, convulsions, or extreme debility ends his sufferings and his life. These terrible symptoms are usually confined to the malignant form of the disease; but inasmuch as the benign polypi may degenerate, as it is termed, and become malignant, no case should ever be neglected, or entrusted to the vis medicatrix naturæ alone.

*Special Symptoms.*—As the prognosis in polypus varies essentially in the different forms of the affection, it is highly important to ascertain the precise character of the tumor before we promise the patient certain relief. And fortunately, the symptoms which characterise each variety are sufficiently obvious to enable us, in most cases, to arrive at a correct diagnosis.

1st. *Vesicular Polypi.*—This form, called by Sir A. Cooper *hydatid polypus*, and by Baron Boyer and others, *mucous polypus*, is distinguished by the tumor being composed of a number of greyish semitransparent vesicles held together by cellular tissue, which contain a watery fluid mixed with mucous, and are so soft as to break from the application of very slight force. In removing them this circumstance is very vexatious; for when you expect to get away the whole mass, you find the forceps containing but a few shreds of cellular tissue; and it is impossible to eradicate the disease by any of the usual operations. The influence of the weather on this kind of polypus is very striking; and as this is not so manifest in the other forms, it should be taken into account in the establishment of the diagnosis. In dry weather the patient is often able to breathe with perfect ease; but in a damp atmosphere, which interferes with the evaporation of the secretion from the tumor, he is almost suffocated by the swelling, which often causes the tumor to protrude from the nostril. In this kind, too, the discharge from the nostrils of mucous is much more copious than in the other forms, and generally, although not invariably, the patient is young, and of a feeble constitution. From the softness of the tumor Boyer supposed that it never displaced the bones of the nose, or, at least, where any displacement occurred it was confined to the vomer. This opinion, however, is not correct, for I have seen great deformity produced by it; and the same circumstance has been observed by others. Some difference of opinion as to the precise nature of these tumors exists among authorities. By Garengeot, Heister, Portal, Hawkins, Watson and others, they are supposed to be nothing more than enlargements of the *mucous follicles* of the lining membrane of the nostril; the enlargement depending upon the obstruction of the duct of the follicle, or upon an increase in quantity or change in quality of the mucous secretion itself. This explanation I am disposed to adopt, from the fact that the little sacs or bags which compose the mass are just the shape of the original follicle, and are entirely distinct from each other. Alibert, and other surgeons of authority, on the other hand, believe the tumors to originate in simple serous infiltration of the submucous cellular tissue; but if this were so, instead of the assemblage of separate and regular bags we should have a single tumor, puffy, and presenting the ordinary appearance of œdema anywhere else.

2d. *Gelatinous Polypi.*—This form has been designated by very different appellations. By Fallopius, Severinus and others, it was called the *nasal hemorrhoid*, from its resemblance to the blind pile. Boyer considered it as one form of *mucous polypus*; others called it *membranous polypus*; by Watson it is termed *indurated polypus*; and by Hawkins *gelatinous polypus*. It is characterised by a consistence somewhat similar to that of hard jelly, and hence its present name; but sometimes it is a little firmer, feeling, when compressed, like a raw oyster. It is usually of a dull white colour, covered by a delicate membrane, from which a yellowish mucous is secreted, generally pyriform in shape, and very brittle. It is this tumor that Alibert has described as the *vesicular polypus*; but, in reality, it is a solid mass of mucous membrane, expanded by effusion into its tissue, and not in separate bags, as in the vesicular form. This secretion becomes thicker, and the tissue containing it more and more solid, until at length the polypus is formed.

The gelatinous polypus is usually the result of inflammation, and appears to be much more local in its character than the vesicular, occurring often in healthy persons, and giving rise to little or no constitutional disturbance, at least for a length of time after its development. It usually occupies the middle meatus, and points forwards.

3d. *Fleshy Polypus.*—This tumor is characterised by its vascularity, florid red colour, slight sensibility, unless irritated, firmness, and extensive attachments. It appears that Celsus describes a tumor of this sort, which, according to him, resembled in consistence and appearance the nipple of the female breast.

It has been also referred to by others as the *fungous polypus*. Similar formations are met with in other mucous cavities, especially the urethra, rectum, and meatus auditorius externus. I have repeatedly encountered this affection in the nostril, and in every case have been able to trace the development of the tumor to previous inflammation or ozena. In the case recently sent me by Dr. Stokes, of Moorestown, ozena had evidently produced the polypus. It appears to me, therefore, that such tumors are composed either of organized or coagulable lymph, secreted by the irritated or inflamed Schneiderian membrane, or of fine granulations which sprout from an ulcerated surface.

In the case of Mrs. T. I removed a large mass of what appeared to be coagulable lymph in a state of partial organization, and beneath it, or rather behind it, I detected a mass of fleshy granulations, which yielded to the pressure of the forceps, and could only be eradicated by caustics.

4th. *Fibrous Polypus.*—This is by far the most formidable of all the benign polypi, in consequence of its firmness and disposition to enlarge. It is, therefore, usually, but especially when of long standing, characterised by more or less displacement of the soft parts and bones of the nostril. It is a firm, solid, and highly organized tumor, consisting of true fibrous tissue, and containing very little fluid in its substance. Its colour is reddish white, or pinkish or brownish; and the delicate smooth membrane that covers its surface is studded here and there with patches of coagulable lymph.

It is almost always attached by a pedicle, even more dense and firm than the tumor itself, which usually, though not invariably, occupies the posterior nares, and points towards, or projects into the fauces.

In most cases there is but one tumor, but we may have several; and lastly, it seems confined to adults —children rarely being thus affected. According to Hawkins, Velpeau and others, this kind of polypus has its origin in the fibrous tissue or periosteum and perichondrium that covers the bones and cartilages of the nose, and lies between them and the mucous membrane proper. There is no doubt, however, that a vesicular, or gelatinous, or fleshy polypus may become fibrous by an extension of the morbid action to the periosteum. Indeed, the only difference in these tumors seems to arise from the tissues first involved.

The vesicular originate in disease of the muciperous follicles. The gelatinous and fleshy in disease of the mucous membrane itself; while the fibrous begins in the periosteum, and the mucous membrane becomes subsequently involved. The fact that a soft polypus may be converted into a fibrous or hard one, is another reason why we should never neglect the immediate treatment of a case, however simple it may appear.

5th. *Hard Polypi.*—Under this head I include those tumors in the composition of which either cartilage or bone largely enters; but few cases of this form of polypus have been reported, and in nearly all it would seem that the disease commenced as a fibrous formation, which subsequently, as is often the case with fibrous tissue elsewhere, becomes converted into cartilage or bone.

(To be continued.)

